# Adaptation and Invasion Dynamics of *Rhipicephalus microplus* in South Africa: Ecology, Resistance, and Management Implications

**DOI:** 10.3390/insects16121204

**Published:** 2025-11-26

**Authors:** Tsireledzo Goodwill Makwarela, Nimmi Seoraj-Pillai, Dikeledi Petunia Malatji, Tshifhiwa Constance Nangammbi

**Affiliations:** 1 Department of Nature Conservation, Faculty of Science, Tshwane University of Technology, Staatsartillerie Rd, Pretoria West, Pretoria 0183, South Africa; seorajpillayn@tut.ac.za (N.S.-P.); nangammbitc@tut.ac.za (T.C.N.); 2 Department of Agriculture and Animal Health, College of Agriculture and Environmental Sciences, University of South Africa, Florida Campus, Roodepoort 1710, South Africa; malatdp@unisa.ac.za

**Keywords:** *R. microplus*, tick invasion, South Africa, acaricide resistance, integrated parasite management, livestock movement, diagnostics (PCR-RFLP, qPCR), climate change, NDVI, wildlife–livestock interface

## Abstract

*Rhipicephalus microplus*, also known as the Asiatic blue tick, is an invasive ectoparasite of significant economic and animal health importance in South Africa. This review synthesises evidence on its ongoing range expansion, which is displacing the native *R. decoloratus*, driven by factors including climate suitability, livestock movement, and communal grazing practices. The tick’s success is compounded by widespread, multi-class acaricide resistance, which has been documented across all major chemical classes. Molecular diagnostics are crucial for accurate surveillance, and population genetics reveal significant gene flow facilitated by livestock movement. Projections indicate that climate change will further expand the tick’s suitable habitat. Current reliance on chemical control is unsustainable. We conclude that an integrated parasite management (IPM) approach combining strategic acaricide rotation, targeted selective treatments, pasture management, and strict biosecurity is critical for sustainable control. Key recommendations include establishing standardised, national resistance monitoring, validating molecular markers for resistance prediction, and developing farmer-facing decision-support tools. This synthesis provides a framework for research and policy to mitigate the impact of *R. microplus* at the wildlife-livestock interface in South Africa.

## Simple Summary

The Asiatic blue tick has recently spread across South Africa, outcompeting the local African blue tick, and causing greater health and financial problems for cattle farmers. These ticks weaken animals by feeding on their blood and can transmit harmful diseases, resulting in reduced milk and meat production. We reviewed published studies on the current distribution of this invasive tick, the factors that facilitate its establishment in new areas, including climate changes, seasonal rainfall, dense vegetation, and cattle movements, and how it can be distinguished from closely related species. We also compiled evidence showing that many populations have become resistant to common tick poisons, which makes them harder to control. Integrated approaches that rotate different chemicals, allow some ticks to survive so that susceptible genes remain, and use pasture rotation and controlled livestock movement were identified as the most promising control strategies. Future challenges include ongoing expansion due to climate change and a lack of coordinated monitoring. We recommend improved nationwide surveillance, farmer education and research into new control tools, including vaccines, to protect cattle health and livelihoods in South Africa.

## 1. Introduction

The invasion and adaptation dynamics of *Rhipicephalus microplus* in South Africa represent a multifaceted concern with significant implications for cattle health, livestock productivity, and public health. This economically critical ectoparasite has spread widely across various regions due to factors such as increased livestock movement, climate change, and agricultural practices that favour tick survival and proliferation [1,2]. The ecological resilience and adaptability of *R. microplus* make it a formidable challenge in pest management. Its ability to transmit pathogens, including noteworthy protozoan parasites such as *Babesia bovis*, poses even greater challenges to livestock health [3,4].

Biologically, *R. microplus* belongs to the hard tick family (*Ixodidae*) and is characterised by a short, straight capitulum, a hexagonal basis capitulum, and an oval scutum [5]. The species is a one-host tick that completes all parasitic stages (larva, nymph, and adult) on a single animal, typically cattle, before dropping off to lay eggs [6,7,8]. Under laboratory conditions, an average female can produce around 1455 eggs, with some variations depending on the host species it is fed upon [9,10]. Its preference for warm, humid habitats explains its high prevalence in tropical and subtropical zones, where environmental factors favour rapid development and host-seeking success [11,12].

Originally endemic to Asia, *R. microplus* has spread globally, including Africa, through trade, livestock movement, and ecological adaptation [13,14]. This tick is responsible for significant economic losses due to its direct and indirect effects on cattle [15,16]. Heavy infestations lead to anaemia, weight loss, hide damage, reduced milk and meat yields, and even mortality in untreated herds. The species is an efficient vector of *Babesia bovis*, *Babesia bigemina*, and *Anaplasma marginale*, which cause bovine babesiosis and anaplasmosis diseases that severely undermine cattle productivity and survival [14]. In addition to these haemoparasites, *R. microplus* has been linked to emerging viral pathogens with potential zoonotic risks [6,17]. Epidemiologically, *R. microplus* demonstrates a high capacity for geographic expansion and replacement of indigenous tick species [18,19]. In South Africa, its spread is particularly concerning because it displaces *Rhipicephalus decoloratus*, a native one-host tick, in key cattle-farming regions. Factors contributing to this displacement include climatic suitability, communal grazing practices, and increased livestock trade and movement [20,21]. The tick’s ability to survive extended dry seasons and to rapidly recolonise areas after rainfall further facilitates its persistence and spread. Climate modelling studies indicate that continued warming trends and changing rainfall patterns may expand its potential range across new provinces [11,22,23,24].

In South Africa, cattle production is a vital economic sector, contributing 25–30% to agricultural Gross domestic product (GDP), yet its market supply is heavily influenced by herd size, a factor evident in the disparity where a large number of small-scale farmers own 40% of the national herd but face productivity challenges in transitioning to commercial systems [25,26,27]. While commercial ranches often have access to veterinary services and acaricides, communal and smallholder farmers who dominate rural production face resource constraints that exacerbate tick burdens [28,29,30]. Limited access to dipping facilities, inconsistent acaricide availability, and knowledge gaps in Integrated Pest Management (IPM) lead to persistent infestations and the development of resistance [31]. Moreover, communal grazing areas provide ideal conditions for tick transmission, facilitating rapid re-infestation cycles among livestock populations. South Africa’s geography and climate play a pivotal role in shaping the distribution and abundance of *R. microplus*. The country’s diverse environment ranges from humid subtropical zones along the east coast to semi-arid savannas and temperate highlands. Warm, moist provinces such as KwaZulu-Natal, the Eastern Cape, and parts of Limpopo offer optimal microclimates for tick proliferation. At the same time, cooler, drier regions of the interior plateau impose natural limits to its establishment [14,32]. Nonetheless, climatic shifts and ecological disturbances such as changes in vegetation cover and interactions between wildlife and livestock are altering these traditional boundaries.

Management implications arising from the invasion of *R. microplus* include the need to develop IPM solutions that combine chemical, biological, and physical control methods. Research on alternative control strategies, such as the use of botanical extracts and the development of genetically engineered vaccines, is increasingly investigated as a way to enhance control approaches while minimising adverse environmental effects [33]. Studies focusing on the immunological responses of cattle and the identification of protective antigens associated with the tick have shown promise for future vaccine formulations, which could significantly improve tick management practices [34,35]. Moreover, understanding the genetic diversity within and among populations of *R. microplus* can provide insights into local adaptations and the mechanisms behind resistance development, thus informing tailored management strategies [36,37]. The potential for horizontal gene transfer plays a role in shaping resistance profiles, particularly concerning the increasing failures of conventional acaricides [38,39]. Thus, molecular investigations into the tick’s biology are crucial for developing innovative approaches to combat this persistent pest.

The socio-economic context surrounding tick management in South Africa also merits attention, as livestock owners frequently face pressures related to financial constraints and knowledge gaps concerning effective tick control practices [40]. Educational initiatives to inform farmers about tick biology, resistance mechanisms, and integrated management options are critical for enhancing compliance and improving livestock health outcomes [40,41]. This review article aims to synthesise current knowledge on the ecology and management of *R. microplus* in South Africa, including invasion history, resistance development, and control strategies, and to identify critical knowledge gaps and future directions.

## 2. Invasion History and Current Distribution in South Africa

**Figure 1:**
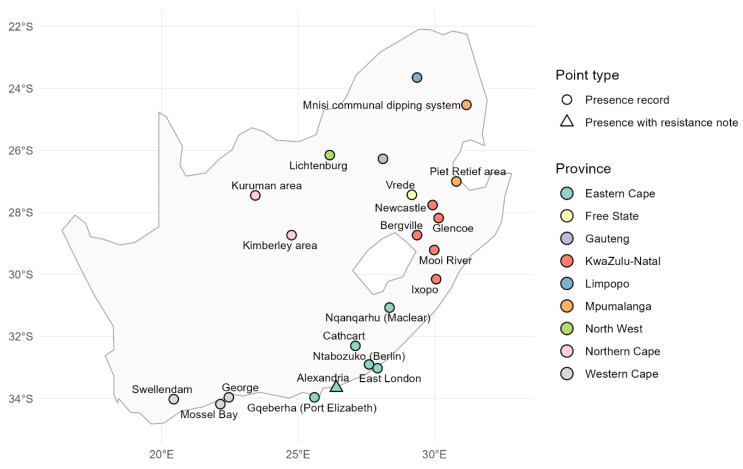
Reported distribution of *R. microplus* across provinces of South Africa (2000–2023 survey data). Created using data from [14,43,54].

The invasion history and current distribution of *R. microplus* in South Africa reflect a complex interplay of ecological factors, human activities, and specific agricultural conditions (see Figure 1 for a map of reported occurrences). The tick, originally from Asia, notably expanded across tropical and subtropical regions, including Africa, through the movement of infested livestock and changes in agricultural practices [42,43]. This pest, known for its role in transmitting various pathogens, including *Babesia* spp., *A. marginale*, has significantly impacted livestock health and economics in farming communities [13,44].

A notable timeline of *R. microplus* presence in South Africa began with its introduction in the 1890s, following the rinderpest epidemic, which resulted in the importation of cattle from affected regions [45]. The first records of the tick’s occurrence indicated its presence in the southern coastal belt of South Africa around 1908 [46]. Over subsequent decades, its distribution spread northward and into the Eastern Cape (EC) Province, and subsequently to the KwaZulu-Natal (KZN) coastal belt [43,46]. By the mid-20th century, reports of *R. microplus* had been documented throughout the EC, with a notable concentration along the coast, where climatic conditions favour tick proliferation [13,47].

Current distribution analysis of *R. microplus* indicates a robust presence across not only the coast but also hinterland provinces, as evidenced by increasing populations in several regions of KZN and EC, particularly in the King Sabata Dalindyebo Municipality, which is indicative of its successful adaptation to inland farming systems [42,48]. The dynamics of tick populations are clearly influenced by environmental factors such as humidity and temperature, making areas like the EC, with its diverse climatic zones, especially conducive to tick development and survival [42,48].

In recent years, strategic surveys have employed molecular techniques to confirm the presence of *R. microplus*, providing geographical insights and confirming that this species has displaced *R. decoloratus* across many regions in South Africa [13,46,49]. The displacement of the indigenous blue tick by the invasive Asiatic cattle tick is well-documented in multiple provinces. In Limpopo Province, a study found that the newly arrived *R. microplus* made up 93.4% of cattle ticks collected, compared to only 6.6% for *R. decoloratus*, and by the end of the study, *R. decoloratus* was virtually absent on communal cattle dips [43]. Similarly, in the Eastern Cape, the tick is now present throughout the coastal region and has been recorded at numerous localities in the central-western region, providing evidence that it is displacing the endemic *R. decoloratus* [13].

The competitive displacement has been attributed to the rapid adaptation and reproductive capabilities of *R. microplus*, enabling it to outcompete endemic tick populations. [48,50]. Key factors driving this displacement include the higher fecundity of *R. microplus*, with a single female laying ~1450 eggs on average, enabling rapid population buildup. Its strong preference for warmer and wetter climatic conditions is also critical, with distribution models showing that its occurrence probability rises sharply above ~23 °C and with greater rainfall [49]. Furthermore, the species often arrives carrying a significant advantage: acaricide resistance. Globally, *R. microplus* has developed resistance to all major classes of acaricides [51], a trend confirmed in South Africa by studies showing the establishment of deltamethrin resistance in field populations. The combination of a rapid life cycle, high reproductive output, specific climatic preferences, and pre-existing acaricide resistance has enabled *R. microplus* to invade and progressively displace *R. decoloratus* [52]. Evidence suggests that within the EC, due to environmental changes and livestock management strategies, *R. decoloratus* populations are declining, leading to significant shifts in tick community structure and dynamics [43,46,53].

The production systems in these areas are primarily focused on cattle farming, which supports both communal and commercial operations. The spread of *R. microplus* is more pronounced in communal grazing areas, whereas its establishment on well-managed commercial farms has historically been limited [46,54]. This disparity is starkly illustrated by a national survey of commercial farms (1998–2001) that found *R. microplus* on only 4 of 184 farms, compared to *R. decoloratus*, which was present on 180 farms [13]. The emergence of *R. microplus* has significant implications for livestock health and productivity in pastoral systems, demanding novel management strategies to mitigate the economic losses associated with tick infestations and associated pathogenic transmissions [13,55]. Current management practices, including chemical interventions, face growing challenges due to the development of acaricide resistance in local tick populations [42,43]. For example, high frequencies of resistance alleles to pyrethroids and amitraz have been found in communal *R. microplus* populations, underscoring this challenge [56,57]. In summary, the combination of a rapid life cycle, high reproductive output, climatic preferences, and pre-existing acaricide resistance has enabled *R. microplus* to invade and progressively displace *R. decoloratus*.

Furthermore, the adoption of integrated pest management strategies that incorporate biological control methods alongside traditional chemical treatments is critical to counteract the adverse impacts of *R. microplus*. Such strategies are geared not only towards reducing tick numbers but also towards minimising ecological disruptions while enhancing the resilience of livestock systems in South Africa [58,59].

## 3. Drivers of Spread and Establishment

The spread and establishment of *R. microplus* in South Africa pose significant challenges for livestock management, influenced by multiple factors including climate, cattle movement, communal grazing practices, vegetation health, and rainfall patterns. This comprehensive understanding of these factors contributes to elucidating the population dynamics and distribution of this ectoparasite.

### 3.1. Climate: Warm, Humid Zones

Temperature and humidity thresholds. Field and laboratory studies indicate that *R. microplus* eggs and larvae develop and survive optimally at temperatures ranging from 15 to 29 °C and high relative humidity. Experiments on off-host larvae reported that minimum relative humidity ≤ 63% reduced survival by 53–72 days; larvae of the closely related *Rhipicephalus australis* survived less than 15 days when relative humidity dropped below 65% and required ≥95% humidity to replenish moisture [10]. Eggs continued developing at 11–21 °C with 69–70% humidity, and survival was prolonged at 15–29 °C with high humidity [10]. Surveys in South Africa found that ticks were collected at temperatures between 12 °C and 35 °C and humidity between 40% and 65%, noting that relative humidity above 70% is optimal for engorged females and eggs [32]. Consequently, coastal provinces such as the Eastern Cape and KwaZulu-Natal, which experience warm temperatures and high humidity, are particularly prone to heavy tick burdens [2,31,60,61].

Climate-change impacts. Climate change is expected to extend the tick’s active season and increase the number of generations per year. A review on climate-driven range shifts noted that temperature and humidity are key determinants of *R. microplus* distribution, and that warming and changing precipitation patterns will alter the tick’s life cycle and abundance [62]. Another analysis argued that macroclimate variables, temperature, humidity, and water vapour deficit, regulate the tick’s survival and development, and suggested that warmer niches can enable the species to spread southwards, if humidity remains adequate [22]. Higher tick densities increase the probability of pathogen transmission to livestock and may create new areas of suitable habitat.

### 3.2. Cattle Movement and Communal Grazing Practices

Several socio-economic and management factors contribute to the spread of *R. microplus* in South Africa. Cattle movement between grazing areas, whether for trade, seasonal pastures, or herd management, increases contact between animals of diverse origins and facilitates the transfer of ticks across regions [63,64]. Communal grazing systems, which are widely practised in rural areas, further intensify this risk by allowing multiple farmers to share unfenced pastures, resulting in the continuous mixing of livestock from different owners and herds [43,65,66,67]. Such practices create repeated opportunities for ticks to move from infested to susceptible cattle, thereby sustaining prominent levels of infestation. The consequences for tick control in these contexts are significant: intermingling cattle populations lead to elevated exposure rates, while the absence of closed farming systems and strict movement regulation makes coordinated tick management strategies difficult to implement. These practices not only complicate control efforts but also shape tick population dynamics, promoting the spread and persistence of invasive species such as *R. microplus* [43,54].

### 3.3. Vegetation Index: NDVI as an Indicator

The increasing prevalence of *R. microplus* in South Africa has driven extensive research into its ecological determinants, particularly the role of vegetation health and environmental factors, as measured through the Normalised Difference Vegetation Index (NDVI). Normalised Difference Vegetation Index serves as a standardised metric of vegetation density and greenness, with direct implications for habitat suitability and tick population dynamics. High NDVI values indicate dense vegetation, which creates favourable microclimatic conditions such as shade, moisture retention, and reduced desiccation stress. These conditions are critical for tick survival and reproduction, especially in areas with high cattle density, where infestations pose significant veterinary and economic challenges [1,46,68,69,70].

Recent evidence demonstrates a strong positive correlation between NDVI and tick prevalence. For example, ref. [53] reported that South African provinces with higher NDVI values experienced increased tick loads in Nguni (*Bos taurus*) cattle. Similarly, Horak et al. [71] documented the dispersal of *R. microplus* in environments characterised by elevated vegetation density, reinforcing the association between vegetation health and tick persistence. These findings highlight NDVI’s utility not only as a retrospective ecological indicator but also as a predictive tool for anticipating tick distributions under changing climatic and ecological conditions [72,73].

Geographical expansion patterns further illustrate this relationship. *R. microplus* has successfully colonised diverse agro-ecological zones across South Africa, often displacing the indigenous *R. decoloratus*. This displacement is particularly evident in the Eastern Cape, where warmer climates and higher NDVI values provide optimal conditions for establishment [44,46]. Increased vegetation density also correlates with higher host availability, which reinforces tick persistence and enhances reproductive success [18]. The displacement of *R. decoloratus* is of particular concern as *R. microplus* is a proven vector of tick-borne pathogens such as *Babesia* and *Anaplasma*, which have severe implications for cattle health and productivity [1,70].

The application of NDVI extends beyond ecological observations to practical agricultural management and policy development. By integrating NDVI into livestock monitoring and pest control programmes, it is possible to identify high-risk areas and implement targeted interventions to mitigate these risks. Vegetation growth patterns mapped through NDVI allow for pre-emptive control measures against emerging tick populations [68,69]. Additionally, climate change is altering precipitation and vegetation dynamics, further influencing NDVI trends. Continued monitoring of NDVI will therefore be essential for adaptive tick management strategies, particularly as rainfall fluctuations directly affect vegetation health and tick habitat availability [1,53,74].

### 3.4. Rainfall Seasonality

The ecological success of *R. microplus* in South Africa is closely tied to the country’s well-defined rainfall seasonality, which governs vegetation health, host availability, and disease risk. Most of the country (including Limpopo, Mpumalanga, KZN, EC, Free State, and parts of North-West) falls in the summer-rainfall zone, with the rainy season running roughly from November to March. In these regions, *R. microplus* larvae hatch and quest soon after the summer rains, and tick burdens on cattle peak in late spring–summer [75]. High rainfall supports lush vegetation that retains moisture and prevents tick desiccation, as reflected in high NDVI values that correlate with elevated tick burdens on livestock [13,71,76]. For instance, in an EC study, the number of adult *R. microplus* on cattle was highest in November (late spring), with larval stages peaking in October and again in February, precisely following the onset of rains [77]. During the prolonged dry winter (June–August), little tick activity occurs; off-host stages desiccate, and on-host ticks dwindle [46,75]. In contrast, prolonged drought reduces vegetation productivity and temporarily suppresses tick populations, though *R. microplus* often rebounds rapidly once moisture returns [78,79]. Seasonal rainfall also synchronises questing activity, heightening encounters with cattle and increasing the risk of babesiosis and anaplasmosis transmission [13,76]. Moreover, favourable rainfall-driven conditions facilitate the displacement of indigenous ticks, further enhancing the invasive success of *R. microplus* [79]. Its resilience to climatic extremes underscores the persistent threat this species poses to livestock health, reinforcing the need for climate-informed surveillance and management strategies.

### 3.5. Ecological Niche and Climate-Change Projections

The ecological niche and climate-driven dynamics of *R. microplus* in South Africa are influenced by a combination of climatic, ecological, and anthropogenic factors. Ensemble species distribution models (SDMs) indicate that *R. microplus* occupies one of the broadest climatic niches among cattle ticks, with warm, humid to sub-humid regions along the eastern escarpment and coastal provinces emerging as core suitability zones [21]. Independent surveys corroborate these predictions, showing that by 2013–2015 the tick was widespread across the EC and had extended into the Northern and Western Cape, often in irrigated pastures that provide moist microclimates. These expansions also coincided with displacement pressure on the native *R. decoloratus* [13]. Across Africa and beyond, SDM studies consistently identify precipitation and temperature variables, such as warm-season rainfall, isothermally, and seasonal temperature ranges, as key determinants of *R. microplus* survival, underscoring its sensitivity to desiccation and preference for stable, humid conditions [80,81]. Additional factors such as vegetation structure, irrigated land use, and host movement through cattle trade and communal grazing further facilitate persistence and dispersal across otherwise marginal landscapes.

In the future, climate change is projected to enhance these dynamics. South Africa-wide projections for 2041–2060, based on CMIP6 scenarios, suggest that *R. microplus* will experience the most significant range increase among cattle ticks, with an estimated 14% national expansion [21]. These shifts raise significant veterinary concerns. Historical and contemporary surveys confirm that *R. microplus* displaces *R. decoloratus*, with hybridisation suggested in some regions [13]. Continued climate-driven expansion heightens the risk of competitive replacement, which is concerning given *R. microplus* is a more efficient vector of *Babesia bovis* and *Anaplasma* spp. Methodologically, ensemble modelling approaches reduce bias, but limitations remain: host density, movement networks, vegetation indices, and irrigation are often underrepresented, suggesting realised spread may outpace purely climatic predictions. Historical data are uneven across provinces and host species; yet recent field campaigns confirm genuine range expansions into new habitats.

## 4. Diagnostic Advances

*Rhipicephalus microplus* and *R. decoloratus* are closely related tick species with minimal and variable morphological differences, making them difficult to differentiate visually. [82]. Accurate identification is critical in South Africa because management and disease risk differ between the two [55]. Standard identification relies on microscopic analysis of subtle morphological features, such as the shape and presence of spurs and setae on the palps. Adults can be distinguished morphologically by anal shields and hypostome dentition, but larval or damaged specimens are hard to separate by eye [19]. These traits, however, can be challenging to observe and interpret accurately, particularly when dealing with immature or damaged specimens [83].

Thus, molecular methods have been adopted. Molecular techniques have been developed to address these limitations. A well-established approach is ITS2-PCR–RFLP: the ribosomal ITS2 region is amplified by PCR and digested with restriction enzymes to yield species-specific band patterns. For example, Lempereur, Geysen and Madder [82] developed an ITS2-PCR–RFLP that reliably distinguishes African Boophilus ticks; this has been used to confirm identifications in South African studies. PCR-RFLP involves amplifying a specific DNA region with primers, followed by digestion with a restriction enzyme. The resulting DNA fragments are separated on a gel, producing species-specific banding patterns that allow clear differentiation between *R. microplus* and *R. decoloratus* [84,85,86,87].

Real-time quantitative PCR (qPCR) assays have also been applied; species-specific primer/probe sets targeting ITS2 or mitochondrial sequences can rapidly screen many tick DNA samples. The advantage of qPCR is its high sensitivity and throughput, which enable the quantification of mixed-species infections in pooled samples. However, it requires specialised equipment and careful primer design to avoid cross-reactions [88,89,90]. Quantitative PCR (qPCR), while not as detailed for distinguishing species, is highly sensitive and can be used to detect and quantify specific tick DNA, making it valuable for presence/absence identification and population studies [13,91,92,93].

Common genetic markers include ITS2 rDNA, which offers high inter-species variability and multicopy sensitivity, making it an excellent choice for species identification (used in the RFLP method above). Additionally, mitochondrial genes (e.g., *16S rDNA* or *COI*) evolve rapidly and often provide clear species “barcodes.” Mitochondrial COI is widely used globally; the South African *R. microplus* group with the standard Clade A COI haplotype [94,95,96,97]. Mitochondrial genes such as Cytochrome Oxidase I (COI) and 16S ribosomal RNA (16S rRNA) are commonly employed, as their conserved nature makes them ideal for phylogenetic studies and accurate differentiation between closely related species [84,91]. While PCR cross-amplification and nuclear mitochondrial pseudogenes (NUMTs) can be a drawback of mtDNA markers, these issues are often addressed in modern molecular studies through careful primer design and amplification strategies [98,99]. In addition, internal transcribed spacer (ITS) regions, particularly ITS2, have been used to develop PCR-RFLP assays for identifying *Rhipicephalus* species, although these may not be as universally effective as mitochondrial markers across all taxa [100].

South African (SA) tick labs primarily rely on morphological identification, supplemented by conventional PCR-RFLP (ITS2) analysis, for distinguishing between the invasive *R. microplus* and the indigenous *R. decoloratus*. While qPCR is faster and more sensitive, its cost limits widespread use, and sequencing is reserved for ambiguous cases or research purposes [84,101,102]. The ability to accurately differentiate the invasive *R. microplus* from the indigenous *R. decoloratus* is crucial in SA because *R. microplus* is the primary vector for the more pathogenic *B. bovis*, a focus of quarantine measures and control strategies. The ongoing displacement of *R. decoloratus* by *R. microplus* in certain regions highlights the need for reliable and accurate identification methods to monitor their distribution and manage disease transmission effectively [103,104]. 

## 5. Acaricide Resistance Landscape

In South Africa, *R. microplus* has rapidly evolved resistance to all major acaricide classes, driven by heavy and often improper use [105,106]. Historically, *R. decoloratus* developed resistance to DDT and organophosphates in the 1970s and 1980s, and to synthetic pyrethroids and amitraz by the 1990s [66]. The invasive *R. microplus* arrived (often on already-resistant cattle) and similarly acquired resistance. Several bioassays are routinely used to evaluate acaricide resistance in *R. microplus*. The Larval Packet Test (LPT) is one of the most widely employed methods, exposing larvae to treated surfaces to measure mortality and determine resistance levels [106,107]. The Adult Immersion Test (AIT) is another standard assay in which engorged female ticks are immersed in acaricide solutions to assess efficacy and detect resistance in adult populations. Other phenotypic approaches include larval immersion tests (LITs) and the more recent larval tarsal test (LTT), both of which provide additional tools for detecting resistance in immature stages [108].

Early surveys in communal Eastern Cape found *R. microplus* populations resistant to cypermethrin and amitraz at some dip sites [55,109]. A more recent genetic study in Mpumalanga’s Mnisi communal area found *R. microplus* populations that were nearly fixed for pyrethroid and formamidine resistance alleles: ~81% carried a pyrethroid-resistance allele and ~55% carried an amitraz-resistance allele; all ticks sampled were homozygous kdr (knockdown) mutants [110,111]. In practical terms, almost no pyrethroid dip now fully controls that population, and the efficacy of amitraz is declining. Acaricide resistance in *R. microplus* arises through a range of mechanisms. Target-site resistance involves genetic mutations that alter the molecular target of the acaricide, thereby reducing its effectiveness; a key example is the knockdown resistance (kdr) mutation in the voltage-gated sodium channel, which confers resistance to pyrethroids [52,112]. For example, the para-type sodium channel gene in SA ticks carries the C190A (I61V) mutation in Domain II, which underlies high-level resistance to synthetic pyrethroids [112,113]. Metabolic resistance occurs when ticks increase their production of detoxification enzymes such as cytochrome P450S, esterases, and glutathione S-transferases, allowing them to break down and eliminate acaricides more efficiently [112,114,115]. Mutations in the octopamine/tyramine receptor (e.g., RmβAOR gene) have been associated with amitraz resistance in *R. microplus* elsewhere and appear to be spreading in SA populations [57,116,117,118].

**Figure 2:**
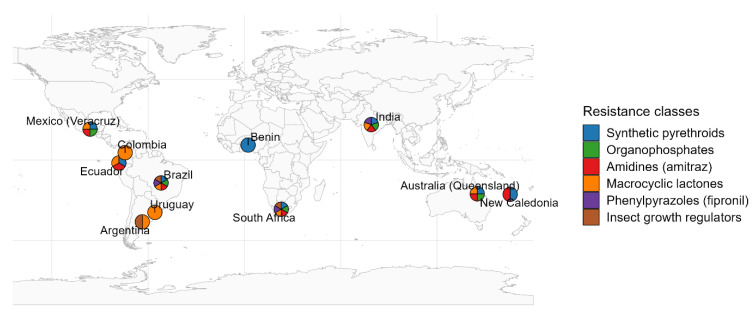
Global distribution of reported *R. microplus* acaricide resistance cases, by chemical class. Created using data from [42,43,51,57,106,112,122,123,124,125,126,127,128,129,130,131,132,133,134,135].

By contrast, a survey of commercial farms found *R. microplus* still mostly susceptible to standard acaricides, while *R. decoloratus* showed higher resistance frequency (66% of populations had cypermethrin resistance, 26% had amitraz resistance) [43,66]. This indicates that *R. microplus* resistance is currently most severe in communal settings where dips are shared among multiple animals. Resistance is now widespread in regions where cattle ticks are prevalent, with notable cases reported in South America (particularly Brazil), India, and South Africa (Figure 2) [106,119]. Table 1 summarises resistance cases recorded to date. In South Africa, studies conducted in provinces such as KwaZulu-Natal and Mpumalanga have documented high levels of resistance and multi-resistance using LPT assays, findings that mirror those from Brazil [106]. South African farmers have tended to rely heavily on a few product classes (cypermethrin and amitraz) for over two decades.

The development of resistance is strongly associated with selective pressure from over-reliance and repeated use of acaricides belonging to the same chemical class [112,120]. To address this, integrated strategies are essential. Integrated Parasite Management (IPM) approaches, which combine chemical, biological, and management-based interventions, are widely recognised as crucial for sustainable tick control [51,121]. Molecular diagnostics are also becoming increasingly important, as the development of reliable genetic markers enables more effective resistance monitoring and can guide evidence-based management decisions [52]. Despite advances in understanding, significant challenges remain. The molecular mechanisms underlying resistance to certain acaricides, such as amitraz, fipronil, and macrocyclic lactones, are still poorly understood. Furthermore, the identification of consistent genetic markers that reliably predict resistance across diverse tick populations remains elusive, highlighting the need for continued research in this area.

## 6. Population Genetics and Adaptation

Population genetic studies of *R. microplus* have provided valuable insights into the tick’s adaptability, dispersal, and evolutionary responses to selective pressures. Molecular markers such as microsatellites, short, repetitive DNA sequences with high variability, have been widely used to assess genetic diversity and population structure, while single-nucleotide polymorphisms (SNPs) allow for high-resolution detection of fine-scale differentiation and adaptive signatures. [136,137,138]. Together, these markers reveal patterns of gene flow, local adaptation, and resistance mechanisms that underpin the remarkable ecological success of *R. microplus*.

Microsatellite analyses have identified distinct genetic clusters while also revealing high within-population variation, suggesting that gene flow, primarily facilitated by livestock movement, plays a significant role in shaping genetic structure across regions. For example, Sungirai et al. [139] reported high genetic diversity (expected heterozygosity, *He*, ranging from 0.755 to 0.802) and low genetic differentiation (F_ST = 0–0.076) among Southern African populations, with 97% of the genetic variance occurring within populations. These findings are consistent with widespread dispersal and limited isolation, a pattern reinforced by the movement of infested cattle across provincial and national borders. In contrast, a newly established population in Matabeleland North, Zimbabwe, exhibited markedly reduced genetic diversity, reflecting a founder effect associated with range expansion [140]. Such founder events highlight how *R. microplus* can colonise new areas despite genetic bottlenecks, with rapid adaptation facilitating persistence under novel ecological conditions.

SNP-based approaches have advanced the detection of resistance-associated loci. A notable example is the identification of a variant in the *RmβAOR* gene linked to amitraz resistance, which provides direct evidence of genetic adaptation to acaricide pressure [141]. These findings emphasise that acaricide resistance in *R. microplus* is not simply a phenotypic outcome but has a heritable genetic basis, complicating control strategies and necessitating molecular surveillance programmes. Mitochondrial DNA markers, particularly cytochrome oxidase I (COI), have further revealed that *R. microplus* is part of a broader species complex. Some lineages, such as those from southern China and northern India, appear genetically closer to *R. annulatus* than to other *R. microplus* clades [18]. This cryptic diversity complicates taxonomy, epidemiological predictions, and resistance monitoring, highlighting the importance of comprehensive phylogeographic analyses. Despite the utility of microsatellites, they present limitations, including the occurrence of null alleles and variability in flanking regions that may affect amplification and interpretation [142]. Nevertheless, population genetic tools remain indispensable for elucidating the spread, adaptation, and resistance of *R. microplus*. Integrating microsatellite, SNP, and mitochondrial data with ecological and epidemiological models will improve our capacity to develop geographically targeted and evolutionarily informed strategies for managing this economically significant pest.

## 7. Integrated Management Approaches for *R. microplus*

Non-chemical and integrated strategies play a vital role in managing *R. microplus* populations and slowing the development of acaricide resistance, moving beyond the current South African practice, which relies mainly on acaricide-based control (dipping/spraying) as its backbone [105]. Commercial farmers typically dip cattle 8–12 times per year with synthetic pyrethroids or amidines, while some use pour-on or injectable acaricides [109]. However, resistance and cost are critical limitations. Overuse of a single acaricide class has selected multi-resistant tick strains, and the quality of communal dips is often poor (weak solutions, dilutions). Compliance and access are issues: many smallholders dip irregularly due to travel or cost, leaving refugia of untreated ticks [105,109].

Pasture rotation is one of the most widely recommended approaches, where grazing paddocks are rotated with sufficient rest periods [143,144]. Adjusting grazing patterns can interrupt tick life cycles. Studies in tropical conditions have shown that resting pasture for a sufficient period breaks the *R. microplus* cycle. For instance, Cruz-González et al. [143] found that a 45-day paddock rest (with no cattle grazing) significantly reduced *R. microplus* loads on calves compared to continuous grazing. This practice prevents continuous exposure of larvae to hosts, while rest times of approximately 45 days can result in starvation or desiccation of larvae and nymphs on pastures. Such practices are increasingly recommended, but they demand enough land and coordination among farmers (often challenging in communal areas). Breeding and host resistance represent another key tactic. Indigenous breeds like Nguni cattle are more tolerant of ticks than European breeds [145,146]. Promoting hardy or crossbred cattle can reduce tick loads and disease; genetic selection for tick resistance is a promising long-term approach to managing these issues. In practice, however, breed replacement is slow and requires farmer education and resources.

The concept of refugia represents another vital strategy, involving the deliberate maintenance of a portion of the tick population in a susceptible state by avoiding intensive treatment [51,147]. Selective treatment, in which only heavily infested animals are treated while others are left untreated, helps preserve a susceptible gene pool within the tick population [148]. Strategic use of “refugia” is conceptually valuable, for example, treating only a portion of cattle or alternating treatments so that a subset of ticks remains unexposed to acaricides, thereby slowing resistance development [147]. Biological control offers a chemical-free option. Entomopathogenic fungi (e.g., *Metarhizium* spp.) have shown promise against cattle ticks in trials [149,150,151]. The South African biopesticide Tickoff^®^, which contains *Metarhizium anisopliae* (isolate ICIPE 7), is currently being evaluated in a randomised controlled field trial in Kenya to test its effectiveness in controlling natural tick infestations and reducing tick-borne diseases in cattle. This study is comparing Tickoff^®^ against a standard chemical acaricide, Triatix^®^, and a placebo, and it is designed to provide data on the bioacaricide’s safety and efficacy for potential large-scale use [152,153]. Biological control can reduce tick populations without the risk of resistance; however, consistency in field conditions can be variable, and application can be laborious. Other IPM tools include strategic treatment intervals and rotation between different chemical classes to mitigate selective pressure [154,155]. Current tick control in South Africa primarily relies on chemical acaricides, though resistance is an increasing problem. Vaccines available in SA for tick-borne diseases are live blood vaccines targeting the pathogens themselves (e.g., African and Asiatic redwater, anaplasmosis, and heartwater), rather than the ticks directly. Research is ongoing to identify new antigens and develop more effective, potentially “personalised” or multi-antigen vaccines tailored to African tick populations, but these are not yet commercially available [156]. 

Each approach has limitations. Resistance undermines chemical control (requiring rotation of products that may be unaffordable or banned). Access and compliance gaps exist: many communal farmers lack reliable access to high-quality acaricides and training; dip tanks may be dilapidated or unused. Pasture rotation is constrained by communal land tenure and grazing rights. IPM integration requires extension support and farmer education that are often insufficient in rural areas. Nonetheless, combining methods is key. For example, integrating limited chemical treatment with grazing management and selective breeding can reduce overall tick pressure and preserve the efficacy of acaricides. Reinforcing seasonal dipping schedules with pasture spelling (so-called “rotational grazing with refugia”) is an emerging recommendation in South African tick control programmes.

## 8. Knowledge Gaps and Future Directions

Despite considerable research on *R. microplus*, several gaps remain that limit effective management of this tick. Resistance monitoring remains inconsistent, with limited geographic coverage and little long-term data on both phenotypic resistance and its genetic basis, particularly in the context of new acaricide compounds [52,112,157]. Similarly, understanding of population dynamics is incomplete, especially concerning the interplay of climate variables, host availability, including both cattle and wild ungulates and pasture management practices across diverse South African contexts [22]. Broader ecological interactions, such as the role of wild ungulates and the influence of climate change on tick ecology, also require further study to inform comprehensive management strategies. Another significant gap lies in farmer engagement, as there is a shortage of accessible, practical tools to support decision-making based on local resistance profiles and environmental factors.

To address these gaps, several research priorities have been identified. First, the development and validation of standardised resistance panels, including larval packet tests and adult immersion tests, are necessary for routine monitoring of multiple acaricide classes across South African provinces [52,55]. Second, longitudinal farm cohorts should be established to track acaricide resistance, tick population densities, and the effectiveness of integrated management strategies over time [55]. Genomic surveillance offers an additional opportunity, with the potential to identify molecular markers associated with resistance, enabling rapid prediction of phenotypic resistance and the monitoring of resistance gene flow within populations [52]. Integrating such surveillance with farmer decision-support tools, combining resistance data, ecological information, and climate trends, would provide practical, evidence-based recommendations for sustainable tick control. Parallel to these efforts, research on acaricide efficacy must continue, focusing on both established and novel compounds, particularly within communal farming systems where resistance often evolves rapidly [42,55]. Finally, investigations into host resistance and the development of effective anti-tick vaccines remain essential for reducing reliance on acaricides and promoting long-term sustainability.

## 9. Conclusions

The Asiatic blue tick, *R. microplus*, is now a significant threat to cattle in South Africa. It has successfully spread to multiple provinces, aided by warm, humid climates, seasonal rains, and the movement of livestock, especially in communal grazing areas. This invasion is exacerbated because the tick is outcompeting the native blue tick and has developed resistance to many common tick poisons. Relying only on chemical treatments is no longer effective. The most effective solution is an integrated approach that combines multiple methods. This involves strategically rotating distinct types of tick poisons to slow down resistance, treating only heavily infested animals to keep some susceptible ticks in the population, and using pasture rotation and movement controls to break the tick’s life cycle. To make this work, South Africa needs to take coordinated action. This includes establishing a standard national system to monitor resistance, developing practical tools to assist farmers in making informed decisions, and enhancing tick identification. Investing in research for new control methods, like vaccines, is also crucial for long-term success. By adopting this comprehensive strategy, the country can protect its livestock, reduce disease, and safeguard the livelihoods of its farmers.

## Figures and Tables

**Table 1:** Documented instances of *R. microplus* acaricide resistance in South Africa.

Year/Period	Province/Region	Locality/Site	Evidence/Method	Classes	Example Actives	Notes	References
2008	Eastern Cape	Communal dip tanks (various)	Larval immersion test (LIT)	AM; SP; OP	Amitraz; cypermethrin; chlorfenvinphos	Resistance at 3/45 (AM), 1/45 (SP), 8/36 (OP)	[43]
2013	Mpumalanga	Unspecified (communal)	LTT/LPT bioassay	SP	(Not specified)	One SP-resistant population	[106]
2006–2017	Eastern Cape	Near Alexandria	LIT (lab submissions)	AM; OP; SP	Amitraz; chlorfenvinphos; cypermethrin	Notable multi-resistant population	[43]
2019–2022	KwaZulu-Natal	Various sites	On-animal field assays + lab	OP; FIP; SP; AM; ML	Chlorfenvinphos; fipronil; deltamethrin; amitraz; ivermectin	Phenotypic resistance observed	[106]
2019–2022	Mpumalanga	Various sites	On-animal field assays + lab	OP; FIP; SP; AM; ML	As above	Phenotypic resistance observed	[106]
2019–2022	Western Cape	Various sites	On-animal field assays + lab	OP; FIP; SP; AM; ML	As above	Phenotypic resistance observed	[106]
2019–2022	Eastern Cape	Various sites	On-animal field assays + lab	OP; FIP; SP; AM; ML	As above	Phenotypic resistance observed	[106]
2023	Eastern Cape	King Sabata Dalindyebo	AIT and LIT	AM; SP	Amitraz; deltamethrin	RR up to ~3 (AM) and ~14 (SP)	[42]

AM = amidine (amitraz), SP = synthetic pyrethroid, OP = organophosphate, FIP = fipronil (phenylpyrazole), ML = macrocyclic lactone.

## Data Availability

No new data were created or analyzed in this study. Data sharing is not applicable to this article.

## Abbreviations

AIT	Adult Immersion Test
AM	Amitraz (formamidine)
*B. bigemina*	*Babesia bigemina*
*B. bovis*	*Babesia bovis*
BVDV	Bovine Viral Diarrhoea Virus
*COI*	*Cytochrome Oxidase I* gene
ECP	Eastern Cape Province
FIP	Fipronil (phenylpyrazole)
IPM	Integrated Pest Management
ITS/ITS2	Internal Transcribed Spacer/ITS2
kdr	Knockdown Resistance
KZN	KwaZulu-Natal
LIT	Larval Immersion Test
LPT	Larval Packet Test
LTT	Larval Tarsal Test
ML	Macrocyclic Lactone
NDVI	Normalised Difference Vegetation Index
OP	Organophosphate
PCR-RFLP	Polymerase Chain Reaction–Restriction Fragment Length Polymorphism
qPCR	Quantitative Polymerase Chain Reaction
*R. australis*	*Rhipicephalus australis*
*R. decoloratus*	*Rhipicephalus decoloratus*
*R. microplus*	*Rhipicephalus microplus*
16S rRNA	16S Ribosomal RNA
SNP	Single-Nucleotide Polymorphism
SP	Synthetic Pyrethroid
SSP5-8.5	Shared Socio-Economic Pathway 5/RCP 8.5
